# Supplementary material for: The Systems Biology Research Tool: evolvable open-source software
Source: BMC Syst Biol. 2008 Jun 29;2:55. doi: 10.1186/1752-0509-2-55 (PMC2446383; doi:10.1186/1752-0509-2-55)
Supplement: Additional file 1 — SBRT Archive. An archive of the current version of the Systems Biology Research Tool. [file 1752-0509-2-55-S1.zip › sbrt-1.4.0/doc/developers_guide/api/sbrt/kernel/util/package-use.html]

Uses of Package sbrt.kernel.util


|  |  |  |
| --- | --- | --- |
| **Overview** \| **Package** \| Class \| **Use** \| **Tree** \| **Deprecated** \| **Index** \| **Help** | |  |
| PREV   NEXT | **FRAMES**    **NO FRAMES**     **All Classes** |  |


---


## **Uses of Package sbrt.kernel.util**

| Packages that use sbrt.kernel.util | |
| --- | --- |
| **sbrt.ext.cplex** | Provides a class that allows communication with CPLEX. |
| **sbrt.kernel** | Provides interfaces to define processes. |
| **sbrt.kernel.fba** | Provides classes and interfaces for creating objects relevant to Flux Balance Analysis. |
| **sbrt.kernel.fba.comp** | Provides classes and interfaces for performing Flux Balance Analysis computations. |
| **sbrt.kernel.fba.proc** | Provides classes and interfaces for executing Flux Balance Analysis processes. |
| **sbrt.kernel.io** | Provides classes and interfaces used to perform kernel-level I/O. |
| **sbrt.kernel.math** | Provides classes and interfaces for creating objects relevant to mathematics. |
| **sbrt.kernel.math.algebra** | Provides classes and interfaces for creating objects relevant to algebra. |
| **sbrt.kernel.math.geom** | Provides classes and interfaces for creating objects relevant to computational geometry. |
| **sbrt.kernel.math.geom.comp** | Provides classes and interfaces for performing geometry-related computations. |
| **sbrt.kernel.math.geom.proc** | Provides classes and interfaces for performing geometry-related processes. |
| **sbrt.kernel.math.graph\_theory.proc** | Provides classes and interfaces for performing graph theoretical processes. |
| **sbrt.kernel.math.stat.proc** | Provides classes and interfaces for performing statistical processes. |
| **sbrt.kernel.util** | Provides classes and interfaces for performing utility functions. |
| **sbrt.kernel.utilities.proc** | Provides classes and interfaces for performing utility processes. |
| **sbrt.shell** | Provides classes used by shell-level software. |
| **sbrt.shell.io** | Provides classes and interfaces for reading and writing files. |
| **sbrt.shell.io.fba** | Provides classes and interfaces for reading and writing files relevant to Flux Balance Analysis. |
| **sbrt.shell.mng** | Provides classes and interfaces for creating process managers. |
| **sbrt.shell.mng.algebra** | Provides classes for creating algebra process managers. |
| **sbrt.shell.mng.comb** | Provides classes for creating combinatorics process managers. |
| **sbrt.shell.mng.fba** | Provides classes and interfaces for creating Flux Balance Analysis process managers. |
| **sbrt.shell.mng.geom** | Provides classes and for creating geometry process managers. |
| **sbrt.shell.mng.graph\_theory** | Provides classes and for creating graph theoretical process managers. |
| **sbrt.shell.mng.stat** | Provides classes and interfaces for creating statistical process managers. |
| **sbrt.shell.mng.utilities** | Provides classes and interfaces for creating mathematical process managers. |
| **sbrt.shell.text** | Provides classes and interfaces for defining formats for basic objects. |
| **sbrt.shell.text.fba** | Provides classes and interfaces for defining formats for objects relevant to Flux Balance Analysis. |

Classes in sbrt.kernel.util used by sbrt.ext.cplex

****UnmodifiableMap****
  
          This class is used to create an unmodifiable view of maps.

Classes in sbrt.kernel.util used by sbrt.kernel

****ProgressMonitor****
  
          This interface is used to represent progress monitors.

Classes in sbrt.kernel.util used by sbrt.kernel.fba

****And****

****BooleanRelation****

****Or****

****UnmodifiableMap****
  
          This class is used to create an unmodifiable view of maps.

Classes in sbrt.kernel.util used by sbrt.kernel.fba.comp

****MutableProgressMonitor****
  
          This interface is used to represent mutable progress montiors.

****ProgressMonitor****
  
          This interface is used to represent progress monitors.

****UnmodifiableMap****
  
          This class is used to create an unmodifiable view of maps.

Classes in sbrt.kernel.util used by sbrt.kernel.fba.proc

****ProgressMonitor****
  
          This interface is used to represent progress monitors.

****UnmodifiableSet****
  
          This class is used to create an unmodifiable view of sets.

Classes in sbrt.kernel.util used by sbrt.kernel.io

****UnmodifiableIterator****
  
          This class is used to create an iterator that cannot modify its
underlying collection.

****UnmodifiableList****
  
          This class is used to create an unmodifiable view of lists.

Classes in sbrt.kernel.util used by sbrt.kernel.math

****UnmodifiableMap****
  
          This class is used to create an unmodifiable view of maps.

Classes in sbrt.kernel.util used by sbrt.kernel.math.algebra

****UnmodifiableSet****
  
          This class is used to create an unmodifiable view of sets.

Classes in sbrt.kernel.util used by sbrt.kernel.math.geom

****UnmodifiableSet****
  
          This class is used to create an unmodifiable view of sets.

Classes in sbrt.kernel.util used by sbrt.kernel.math.geom.comp

****UnmodifiableMap****
  
          This class is used to create an unmodifiable view of maps.

****UnmodifiableSet****
  
          This class is used to create an unmodifiable view of sets.

Classes in sbrt.kernel.util used by sbrt.kernel.math.geom.proc

****ProgressMonitor****
  
          This interface is used to represent progress monitors.

Classes in sbrt.kernel.util used by sbrt.kernel.math.graph\_theory.proc

****ProgressMonitor****
  
          This interface is used to represent progress monitors.

Classes in sbrt.kernel.util used by sbrt.kernel.math.stat.proc

****ProgressMonitor****
  
          This interface is used to represent progress monitors.

Classes in sbrt.kernel.util used by sbrt.kernel.util

****AbstractProgressMonitor****
  
          This abstract class is a skeleton implementation of `ProgressMonitor`.

****BooleanRelation****

****MutableProgressMonitor****
  
          This interface is used to represent mutable progress montiors.

****ProgressMonitor****
  
          This interface is used to represent progress monitors.

****UnmodifiableCollection****
  
          This class is used to create an unmodifiable view of collections.

****UnmodifiableSet****
  
          This class is used to create an unmodifiable view of sets.

Classes in sbrt.kernel.util used by sbrt.kernel.utilities.proc

****ProgressMonitor****
  
          This interface is used to represent progress monitors.

Classes in sbrt.kernel.util used by sbrt.shell

****UnmodifiableMap****
  
          This class is used to create an unmodifiable view of maps.

Classes in sbrt.kernel.util used by sbrt.shell.io

****UnmodifiableCollection****
  
          This class is used to create an unmodifiable view of collections.

****UnmodifiableMap****
  
          This class is used to create an unmodifiable view of maps.

****UnmodifiableSet****
  
          This class is used to create an unmodifiable view of sets.

Classes in sbrt.kernel.util used by sbrt.shell.io.fba

****UnmodifiableMap****
  
          This class is used to create an unmodifiable view of maps.

****UnmodifiableSet****
  
          This class is used to create an unmodifiable view of sets.

Classes in sbrt.kernel.util used by sbrt.shell.mng

****UnmodifiableSet****
  
          This class is used to create an unmodifiable view of sets.

Classes in sbrt.kernel.util used by sbrt.shell.mng.algebra

****UnmodifiableSet****
  
          This class is used to create an unmodifiable view of sets.

Classes in sbrt.kernel.util used by sbrt.shell.mng.comb

****UnmodifiableSet****
  
          This class is used to create an unmodifiable view of sets.

Classes in sbrt.kernel.util used by sbrt.shell.mng.fba

****And****

****Or****

****UnmodifiableMap****
  
          This class is used to create an unmodifiable view of maps.

****UnmodifiableSet****
  
          This class is used to create an unmodifiable view of sets.

Classes in sbrt.kernel.util used by sbrt.shell.mng.geom

****UnmodifiableSet****
  
          This class is used to create an unmodifiable view of sets.

Classes in sbrt.kernel.util used by sbrt.shell.mng.graph\_theory

****UnmodifiableSet****
  
          This class is used to create an unmodifiable view of sets.

Classes in sbrt.kernel.util used by sbrt.shell.mng.stat

****UnmodifiableMap****
  
          This class is used to create an unmodifiable view of maps.

****UnmodifiableSet****
  
          This class is used to create an unmodifiable view of sets.

Classes in sbrt.kernel.util used by sbrt.shell.mng.utilities

****UnmodifiableSet****
  
          This class is used to create an unmodifiable view of sets.

Classes in sbrt.kernel.util used by sbrt.shell.text

****And****

****Or****

Classes in sbrt.kernel.util used by sbrt.shell.text.fba

****And****

****Or****

---


|  |  |  |
| --- | --- | --- |
| **Overview** \| **Package** \| Class \| **Use** \| **Tree** \| **Deprecated** \| **Index** \| **Help** | |  |
| PREV   NEXT | **FRAMES**    **NO FRAMES**     **All Classes** |  |


---
